# Fibronectin (FN) cooperated with TLR2/TLR4 receptor to promote innate immune responses of macrophages via binding to integrin β1

**DOI:** 10.1080/21505594.2018.1528841

**Published:** 2018-10-13

**Authors:** Dongsheng Fei, Xianglin Meng, Wei Yu, Songlin Yang, Ning Song, Yanhui Cao, Songgen Jin, Lina Dong, Shangha Pan, Mingyan Zhao

**Affiliations:** a Department of ICU, the First Affiliated Hospital of Harbin Medical University, Harbin, China; b Department of Ultrasound in Obstetrics and Gynecology, The First Affiliated Hospital of Harbin Medical University, Harbin, China; c The Key Hepatosplenic Surgery Laboratory, Department of General Surgery, The First Affiliated Hospital of Harbin Medical University, Harbin, China

**Keywords:** Fibronectin (FN), integrin β1, toll-like receptor, phagocytosis of macrophages, innate immunity, pulmonary infection disease

## Abstract

Macrophages could adhere to extracellular matrix molecules(ECM) to induce the expression of pro-inflammatory mediators and phagocytosis that contribute to the pathogenesis of pulmonary infection diseases. Fibronectin (FN) is a large glycoprotein capable of interacting with various ECM molecules produced by a variety of cell types and involved in cell attachment and chemotaxis. However, it is unknown whether FN regulates the expression of pro-inflammatory mediators and phagocytosis of macrophages in the injured lung tissue. Here, we investigated the interaction between FN and integrin β1 in macrophages, which promotes toll-like receptor 2/4 (TLR2/TLR4) signaling pathways to enhance expression of pro-inflammatory mediators and phagocytosis by macrophages. Our results show that lipopolysaccharide (LPS), lipoteichoic acid (LTA) and peptidoglycan (PGN) significantly increase FN expression of macrophages; FN substantially enhances interleukin 6 (IL-6), tumor necrosis factor-α (TNFα), ras-related C3 botulinum toxin substrate 1/2 (Rac1/2), and cell division control protein 42 homolog (Cdc42) expression and phagocytosis of macrophages. However, FN could not enhance pro-inflammatory cytokines and phagocytosis of macrophages induced by LPS and PGN in integrin β1-/- macrophages. Furthermore, applied integrin β1 blocking peptide abrogated the effects that FN promotes innate immune responses of macrophages to LPS and PGN. Those data indicated that the enhanced pro-inflammatory mediators and phagocytosis of macrophages by FN-integrin β1 signal was through co-operating with TLR2/TLR4 signaling. This study suggests that FN play an essential role in the pathogenesis of pulmonary infection disease.

## Introduction

Acute Respiratory Distress Syndrome (ARDS) occurs in approximately 10% of the patients in Intensive Care Unit (ICU) and 23.4% of the ARDS patients requiring mechanical ventilation that is associated with mortality rates of 34.9–46.1% [1]. Pharmacological interventions targeting specific molecules or pathways have not significantly affected actual mortality [2,3]. ARDS pathogenesis is complicated, which can be caused by both direct and indirect insults. The lungs are usually the first organ involved in the inflammatory response in ARDS. Also, the activation of mononuclear macrophages plays a critical role in the inflammatory response. In the early stages of lung injury, alveolar macrophages are activated to release polymorphonuclear neutrophils (PMN) activation factors such as interleukin-1β (IL-1β), interleukin 6 (IL-6), interleukin-8 (IL-8), and tumor necrosis factor-α (TNF-α), which lead to alveolar epithelial cell injury and apoptosis.

Host ECM components could not only adhere microbial pathogen surface molecules to establish an active bacterial infection but also serve as a like-ligand or associated-activated molecules to activate macrophage through their receptors in innate immunity. Those conserved surface molecules of microbial pathogens were named pathogen-associated molecular patterns (PAMPs), which bind to highly conserved receptors on macrophages called pattern-recognition receptors (PRRs), such as toll-like receptors (TLRs), to activate macrophages and dendritic cells. PAMPs on the cell surface of microbial pathogens mainly contain lipopolysaccharide (LPS) in Gram-negative bacteria, lipoteichoic acid (LTA) in Gram-positive bacteria, and mannans (MAN) in yeast, which were recognized TLR4 and TLR2. Other TLRs have been identified to bind double-stranded RNA (TLR3) [4], bacterial flagellin(TLR5) [5], mycoplasmal macrophage-activating lipopeptide-2 kDa (TLR6) [6] and CpG bacterial DNA (TLR9). The initial signal of those receptors will enhance phagocytosis of macrophages and promote pro-inflammatory responses [7]. Also, there also are mannose-binding lectin (MBL), C-reactive protein and serum amyloid protein of the extracellular pattern-recognition molecules [8]. Some molecules released from the injured tissues can also bind to TLRs to elicit immune responses. However, the exact mechanisms of TLR recognition of those ECM molecules remain unclear.

Fibronectin (FN) has been reported to activate macrophages, mast cells, and T cells to release TLR4 dependent cytokine [9–11] . It has been determined that the first Type III (III-1) domain of FN (FnIII-1c) activate TLR4 and TLR2 mediated cytokine release from macrophages and lung fibroblasts, respectively [12]. FN induced the proinflammatory cytokines through activating nuclear factor kappa-light-chain-enhancer of activated B cells (NF-κB) and P38 mitogen-activated protein kinases (p38 MAP kinase) [13,14]. Although FN binds to several integrins, FN is mainly recognized by integrins α5β1 [15] which are also expressed in macrophages and dendritic cells. Recently, it was determined that Mindin, an ECM molecule extensively expressed in various cells, can promote the pro-inflammatory cytokine production and phagocytosis to microbial pathogens through regulating ras-related C3 botulinum toxin substrate 1/2 (Rac1/2) and cell division control protein 42 homolog (Cdc42) [16]. It was also reported that integrin β1^-/-^ macrophages have defective phagocytosis and phagosome formation through down-regulation of guanosine triphosphate hydrolase (GTPase) small proteins, as Rac1/2 and Cdc42 [17]. Also, integrin α5β1 can activate the NLR family pyrin domain containing 3 (NLRP3) inflammasome by direct interaction with a bacterial surface protein [18]. However, it is unclear how FN enable or promote TLR signals to regulate innate immune responses of macrophages. Here we investigated whether FN enhanced TLR2 and TLR4 singling of macrophage activation to enhance phagocytosis, phagosome formation, and pro-inflammatory cytokines production in lung inflammatory diseases.

## Materials and methods

### Mice

Integrin β1(cluster of differentiation 29,CD29) conditional-null “flox” (CD29f/f) mice were purchased from The Jackson Laboratory, USA. We crossed integrin β1CD29f/f mice with LysM-Cre transgenic mice to generate integrin β1^-/-^ mice. All other mice were obtained from Vital River Bioscience CO., Ltd, (Beijing, China), and maintained in the animal facility at the First Affiliated Hospital of Harbin Medical University Vivarium. All animal procedures followed the protocols which were approved by the Institutional Animal Care and Use Committee, the First Affiliated Hospital of Harbin Medical University. All mice used in this study was at 6–12 weeks of age.

### Reagents

Anti-CD11b-phycoerythrin (PE) cyanin 5 or fluorescein isothiocyanate (FITC), CD29-FITC, CD11b-FITC, hCD2 (hCD2)-FITC and CD18 from eBioscience, hCD163-Violet 421 and anti-mouse CD11b-violet 421, were obtained from Biolegend. mAbs against Rac1, Rac2, Cdc42 were purchased from Santa Cruz Biotechnology. Anti-FN antibody (ab2413) and anti-FN antibody [IST-9] (ab6328) were obtained from Abcam. LPS and PGN were purchased from Sigma. We purchased LTA (LTA-SA: Lipoteichoic acid from Staphylococcus aureus, Catalog# tlrl-slta), Man (LM-MS: Lipomannan from Mycobacterium smegmatis, Catalog# tlrl-lmmsl) and Zym (Zymosan: cell wall from Saccharomyces cerevisiae, Catalog# tlrl-zyn) from InvivoGen. ITGB1/Integrin β1/CD29 Antibody Blocking Peptide (β1BP) LS-E29653 (Cat No: LS-E29653; LifeSpan BioSciences, Inc). All of the TaqMan probes were purchased from Applied Biosystems. We purchased recombinant human FN protein (Cat No: ab158459) and natural mouse FN protein (Cat No: ab92784) from Abcam Biotechnology company.

### Mice challenged by LPS

Male (age-matched from 8–12 wk) C57BL/6 mice (Beijing Vital River Laboratory Animal Technology Co., Ltd.) were utilized in this study. All experimental procedures were approved by the ethical committee of the Harbin Medical College (Heilongjiang, China), and conducted by all state regulations. To induce a sepsis model for experimental procedures, *Escherichia coli* LPS (serotype 0111: B4, Sigma-Aldrich, St. Louis, MO, USA, 20mg/kg) was diluted in saline and injected via intraperitoneal injection (IP). Control mice were intraperitoneally injected with saline only. 24 hours after LPS or saline administration, the mice were injected intraperitoneally pentobarbital, lung tissue was harvested and fixed by formalin for Immunohistochemistry (IHC).

### Macrophage phagocytosis

Bone marrow-derived macrophages (BMMϕ) generated from bone marrow were followed by the previous method [19]. Briefly, bone marrow cells of CD29(integrin β1) f/f, CD29(integrin β1) f/f LysM-Cre or C57BL/6 mice were in DMEM containing 20% FBS and 30% L929 cell-conditioned medium for seven days. Integrin β1^-/-^ macrophages were sorted by flow cytometry (FACS). For confocal microscopy, BMMϕs were cultured on sterile glass coverslips in Petri dishes for 18 hours to allow a confluent monolayer to establish.

Peritoneal macrophages from peritoneal cavity were followed by the previous protocol [19]. 1 ml of 3% Thioglycollate medium was injected into the peritoneal cavity for five days and collected the cells from the peritoneal cavity with 5 ml of PBS. Then, the harvested cells were plated in a petri dish for 30 min and removed the suspended cells. The adhesive cells, which is peritoneal macrophages, were collected and counted. Macrophage phagocytosis assay was performed as previously described [20]. To analyze Fc gamma receptor (FcγR)-mediated phagocytosis, macrophages were cultured on glass coverslips for 12 hours, then add IgG-coupled latex beads (diameter 3μM; Sigma-Aldrich). For analysis macrophage phagocytosis to bacteria, *E. coli* conjugated with Fluorescein isothiocyanate (FITC) treated with normal mouse serum was mixed with macrophages (macrophages: FITC conjugated *E. coli *= 1:100) and incubated for 30 minutes at 37°C. Unbound Macrophages were washed with PBS and quenched with trypan blue (2 mg/ml). To remove unbound FITC-*E. coli*, they were fixed with 3.7% paraformaldehyde. Finally, the samples were analyzed by FACS using a BD Biosciences FACScan.

### RNA isolation and real-time quantitative RT-PCR (qPCR)

RNA was extracted using the RNeasy kit (QIAGEN). cDNA was prepared using reverse transcription reagents from Applied Biosystems. qPCR was performed in triplicate with Taqman qPCR Master Mix in the HT7900 ABI Sequence Detection System (Applied Biosystems). Taqman®-based primer/probe assays for IL6 (Mm00446190_m1), TNFα (Mm00443258_m1), and FN (Mm01298063_m1) were purchased from Applied Biosystems. Gapdh (Mm99999915_g1) was used for control.

### Cytokine assays by enzyme-linked immunosorbent assay (ELISA)

Peritoneal macrophages were induced from C57BL/6 mice by 3% Thioglycollate medium for five days. Peritoneal macrophages were prepared in 2 × 10^6^ cells/ml with RPMI 1640 complete medium and plated in 2 × 10^6^ cells/ml/well in 24 well culture plate. Macrophages were treated with LPS, PGN, LTA, Man, and Zym for 4 hours, and the supernatant was assayed for IL-6 and TNFα using an ELISA kit (eBioscience).

### Immunofluorescent staining of mouse and human lung tissue

Lung tissue remote to the cancer nest (physiologically normal lung) and inflamed lung tissue were obtained from patients who underwent surgical treatment at Harbin Medical University Cancer Hospital between January 2006 and December 2007. All of the specimens were obtained under written consent from each patient. This study was approved by the Research Ethics Committee of Harbin Medical University. Formalin-fixed, paraffin-embedded tissue sections from mice and human lung cancer tissue were fixed in methanol with 1 % H2O2 for 15 min at 4° C to reduce nonspecific staining. 0.8% of Triton X100 in phosphate-buffered saline (PBS) was used to permeabilize for 10 min at room temperature. The slides were washed with PBS two times. After lung sections were blocked with blocking buffer, the slides were immunostained with Alex 488 labeled anti-mouse or human FN (1:200), anti-human CD163-Violet 421 (1:200) and anti-mouse CD11b Violet 421 (1:200), nuclear was stained with SiR-DNA-red for two hr at room temperature. The lung sections were imaged using a confocal microscope (LSM 710, Carl Zeiss).

### Quantitative assessment of co-localization of internalized bacteria and lysosomes

Quantitative assessment of co-localization of internalized bacteria and lysosomes was performed by previous method [17]. Peritoneal macrophages were cultured on glass coverslips for 18 hours. Peritoneal macrophages were stained with Lysotracker Red (Molecular Probes) for 3 hours at 37°C, then continue to culture in fluor-another 2 hours. Macrophages were mixed with FITC-conjugated *E. coli* at a ratio of 1:50 (cell/bacteria) for 20 minutes at 37°C. Cell surface-bound bacteria were quenched with trypan blue (2 mg/ml). After incubation periods, macrophage samples were fixed with 3.7% paraformaldehyde after cultured for 25, 30, 35, 40, 45, 50, 55, 60, 65,70, 75, 80, 85 and 90 minutes at 37°C or a fixed time. To determine the kinetics of phagosome maturation, the co-localization of AF488-labeled *E. coli* with Lysotracker Red (a marker of acidic compartments) was examined by confocal microscopy (LSM 710, Carl Zeiss) at every minute interval from mixing macrophages with FITC-conjugated *E. coli* plus LPS and FN or PGN and FN up to 50 min. The percentage of *E. coli*-containing phagosomes that co-localized with Lysotracker (fluorescence overlay) was quantitatively analyzed using the co-localization dialogue of Metamorph software version 7.0 (Universal Imaging, a subsidiary of Molecular Devices) by randomly scanning > 10 cells in each test group in 3 independent experiments.

### Fluorescence resonance energy transfer (FRET) assay

FRET assay was followed as previous protocol [17]. Peritoneal macrophages were incubated glass coverslip at 37°C in the flour-free medium for 18 hours. AF488 conjugated TLR4 Ab or AF488-conjugated TLR2 Ab, and AF594-conjugated integrin β1 Ab was incubated with macrophages at a dilution of 1:200. After incubating for 20, 30, 60 and 120 minutes at 37°C, before microscopy, cells were fixed with 3.7% paraformaldehyde and preserved in 80% glycerol. FRET between AF488 (donor) and AF594 (acceptor) molecules was established using laser confocal microscopy (Leica SP2) and the Leica FRET Acceptor Bleaching wizard, by noting the loss of intensity of donor that accompanies the close molecule proximity of acceptor. Donor fluorescence intensity was recorded in the same sample before and after destroying the acceptor by selectively photobleaching in a carefully defined region of interest. The energy transfer efficiency was calculated as follows: FRETeff = (Dpost − Dpre)/Dpost, where Dpost is the fluorescence intensity of the donor after acceptor photobleaching, and Dpre the fluorescence intensity of the donor before acceptor photobleaching. The FRETeff is considered positive when Dpost > Dpre. The percentage of FRET-positive regions and FRET efficiency values were quantified by randomly counting over ten areas within one cell and counting > 10 cells in each test group in two or more independent experiments.

### Western blot

Western blot was performed using a standard protocol. Briefly, peritoneal macrophages treated with LPS, PGN and LTA were harvested and lysed in cRIP lysis buffer (150 mM NaCl, 1.0% NP-40, 0.5% sodium deoxycholate, 0.1% sodium dodecyl sulphate (SDS), 50 mM Tris-HCl, pH 8.0 and protease inhibitors). The lysates were run by 12% SDS-PAGE gel and transferred onto nitrocellulose membrane. The membranes were blocked with 5% BSA, incubated sequentially with primary and secondary antibodies, and then washed. FN, Rac1, Rac2, and Cdc42 proteins were detected by enhanced chemiluminescence (ECL) detection reagent and quantified by ImageJ software.

### Statistical analysis

Data are presented as means ± sem. Statistical comparisons among treatment groups were performed by randomized-design two-way ANOVA, followed by the Newman-Keuls post hoc test for more than two groups, or by unpaired Student’s t-test for two groups using Prism software (Graph Pad Inc., La Jolla, CA), as appropriate. Statistical significance was defined as a P value of less than 0.05.

## Results

### FN was highly expressed in macrophages in inflammatory response

**Figure 1.**
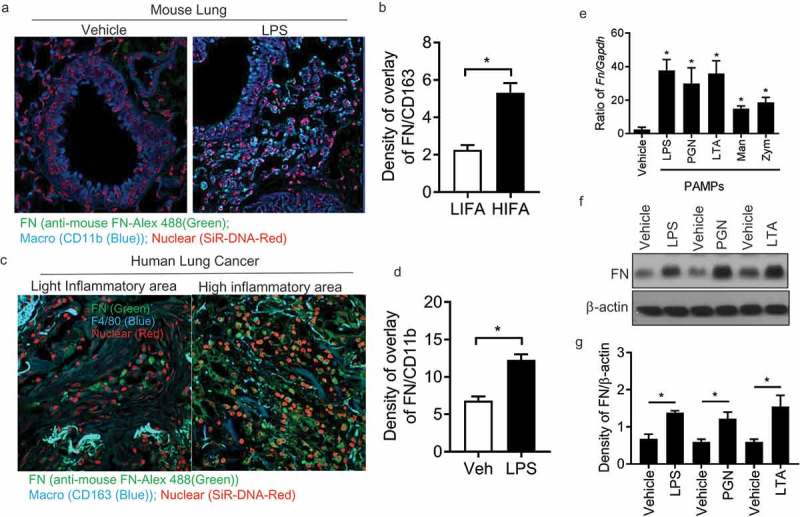
Macrophages express FN during inflammatory responses. Mouse lung tissues after LPS treatment were stained with nuclear SiR-DNA-Red, anti-FN Ab-Alex 488 and Anti-CD11b-APC and observed with confocal microscopy (a) and quantification of FN expression in confocal images of LPS treated lung compared with vehicle-treated lung (b) (n = 11,*p < 0.05). Expression of FN in macrophages around human lung cancer (c) and quantification of FN expression in confocal images of LPS treated lung compared with vehicle-treated lung (d) (n = 11,*p < 0.05). Human lung cancer tissues were stained with SiR-DNA-Red, anti-FN Ab-Alex 488 and Anti-CD163-Violet 421 and observed with confocal microscopy (x 40); *FN mRNA* level of peritoneal macrophages was measured after PAMPs stimulation by q-PCR. (n = 5, *p < 0.05). Briefly, 1 × 10^6^ peritoneal macrophages/well (24 well culture plate) were cultured with LPS (1ug/ml), PGN (10 ug/ml), LTA (5 ug/ml), Mannan (5 ug/ml) and Zym (10 ug/ml) for 4 hours at 37oC. The cell samples were collected with Easy RNA kit and cDNAs were prepared by high-efficiency cDNA kit. FN was quantified by TaqMan real-time PCR system with Taqman FN probe (e); FN expression of peritoneal macrophages was analyzed by western blot After LPS, PGN, and LTA (f), and quantification of the western blot was completed with ImageJ software (g). (n = 3, *p < 0.05). Statistical comparisons among treatment groups were performed by randomized-design two-way ANOVA, followed by the Newman-Keuls post hoc test for more than two groups, or by unpaired Student’s t-test for two groups using Prism software (Graph Pad Inc., La Jolla, CA), as appropriate. Statistical significance was defined as a P value of less than 0.05.

It was determined that FN was extensively produced many types of cells, which not only play a critical role in mammalian development [21] but also interact with other cells to regulate cell adhesion, migration, growth, and differentiation [22–24]. However, the specific effects of macrophage-derived FN remained unclear in regulating innate responses of macrophages in lung inflammatory diseases. Therefore, we firstly examined FN expression in lung macrophages in mice treated with or without LPS stained with DNA-Red, anti-FN Ab-Alex 488 and Anti-CD11b-Violet 421 by confocal microscopy (Figure 1(a)). LPS-treated lung macrophages have significantly increased FN expression compared with vehicle-treated lung (Figure 1(b)). This data suggests that FN may regulate macrophage function in lung inflammation (Figure 1(a)). Also, when frozen human lung cancer tissue slides were stained with anti-mouse FN-Alex 488(green), anti-human CD163 (blue) and nuclear dye-SiR-DNA(red), we found that there had significantly increased FN expression in the area where macrophages were clustered in the inflammation areas around lung cancer (Figure 1(c,d)). We quantified FN expression in mouse lung tissues and human lung cancer tissues as densities of overlay density of FN(green) and CD11b(Violet) (Figure 1(b)) in mouse lung or overlay density of FN(green) and CD163(blue) (Figure 1(d)) in human lung cancer tissue.

To examine whether PAMPs regulated FN, peritoneal macrophages were induced by 3% Brewer thioglycollate medium and collected in 4 – 5 d. Macrophages were stimulated with various PAMPs (LPS, LTA, PGN, Zym, and Man) for 4 hours and RNA was isolated and analyzed by q-PCR. FN mRNA was readily detected in macrophages (Figure 1(e)) and was significantly increased after LPS, LTA and PGN. The result of Western blotting indicated that FN was expressed in macrophages, and FN expression was significantly increased after stimulated with PAMPs (Figure 1(f,g)). These data showed that FN is not only produced in macrophages but also considerably increased after the stimulation of PAMPs, which suggested that FN might be involved in macrophage function.

### FN significantly enhances the proinflammatory response of macrophages to PAMPs *in vitro*

**Figure 2.**
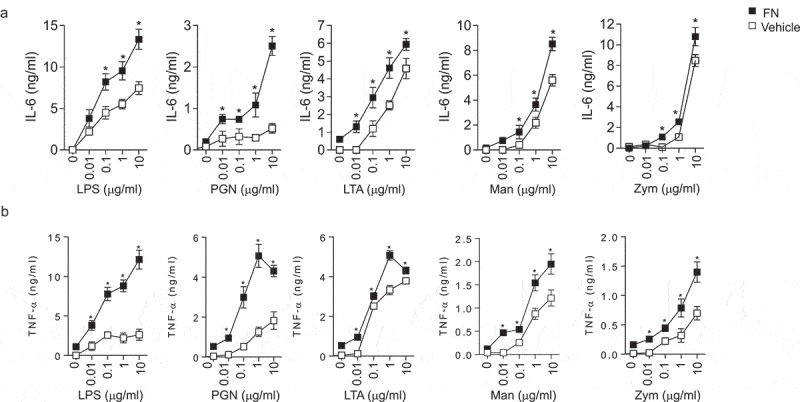
FN enhanced IL-6 and TNFα responses of macrophages to PAMPs. Peritoneal macrophages (2x10^6^ cells/well) from C57L/B6 mice were cultured in 24 well culture plate, and the cells were stimulated with LPS, PGN, LTA, Man and Zym for four h. The supernatants were collected and assayed for TNFα (a) and IL-6 (b) production with ELISA kit. Data are means of triplicates. (n = 3, *p < 0.05). Statistical comparisons among treatment groups were performed by randomized-design two-way ANOVA, followed by unpaired Student’s t-test for two groups using Prism software (Graph Pad Inc., La Jolla, CA). Statistical significance was defined as a P value of less than 0.05.

Macrophages are the first line of defense against the pathogens by a series of biological functions such as pro-inflammatory cytokine production, neutrophil chemotaxis and phagocytic to resist invasion and maintain hemostasis. To evaluate the effects of FN in activating macrophages, the proinflammatory cytokines released by peritoneal macrophages were examined after stimulated with different PAMPs (LPS, PGN, LTA, Zym, and Man) from Gram-positive, Gram-negative bacteria, and yeast to in the presence or absence of FN. Our results indicated that IL-6 (Figure 2(a)) and TNFα (Figure 2(b)) of macrophages stimulated with those PAMPs were dramatically enhanced the expression of these proinflammatory cytokines when FN was presented, which suggested that FN significantly promoted the innate immune response of macrophages to a broad spectrum of pathogens.

### FN dramatically promotes phagocytosis of macrophages to bacteria

**Figure 3.**
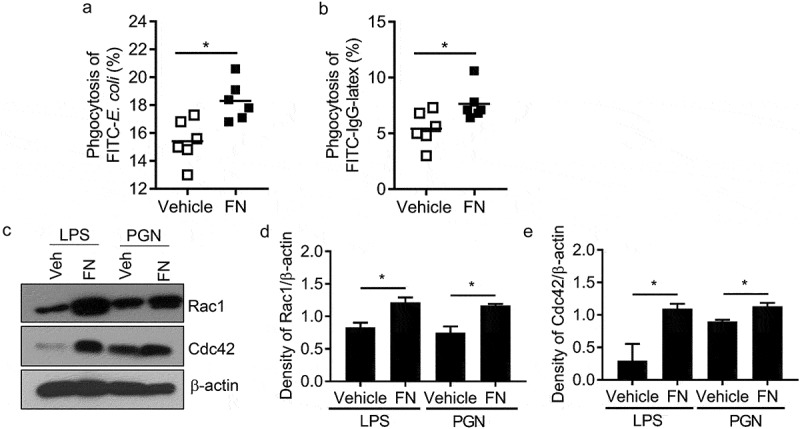
FN functions as an opsonin for macrophage phagocytosis. The effect of FN in macrophage phagocytosis to FITC conjugated E. coli was analyzed by FACS (a). Briefly, peritoneal macrophages from WT mice and bacteria were mixed in suspension at a ratio of 1:10 (cell: bacteria) with and without FN. FITC-E. coli positive cell of macrophages was analyzed by FACS (a); FcγR-mediated phagocytosis of macrophages was also analyzed by FITC-IgG latex beads with/without FN (b). Peritoneal macrophages were cultured on glass coverslips and incubated with IgG-coupled latex at a ratio of 1:10 for 15–30 min at 37°C to assay phagocytosis. Phagocytosis was assessed by detecting the percent of the FITC^+^ cells by FACS. n = 6. ∗, p < 0.01. FN promotes expression of total Rac1 and Cdc42 proteins in peritoneal macrophages after stimulation with LPS and PGN, as determined by Western blot analysis (c). The cells lysates were fractionated by 12% SDS-PAGE gel. Expression of actin serves as a loading control and density of Rac1/β-actin and (d) Cdc42/β-actin (e) was quantified with Image J software. (n = 3, *p < 0.05). Statistical comparisons among treatment groups were performed by randomized-design two-way ANOVA, followed by unpaired Student’s t-test for two groups using Prism software (Graph Pad Inc., La Jolla, CA). Statistical significance was defined as a P value of less than 0.05.

To examine the effect of FN in relating phagocytosis, we used peritoneal macrophages completed phagocytosis assay to bacteria, e.g. *E. coli* and IgG-coated beads in the presence or absence of FN. Our results showed that FN significantly enhanced phagocytosis of macrophages to *E. coli* (vehicle vs. FN: 15.4 ± 1.41% vs. 18.3 ± 1.28%) (Figure 3(a) and Suppl. Figure 1(a)), which indicated that FN could enhance macrophage phagocytosis to some pathogens. Many phagocytoses of macrophages to microbial pathogens was mediated by Fc receptor (FcgR). To evaluate the role of FN in FcgR -mediated phagocytosis, we added recombinant FN to the mixture of macrophages and IgG beads to examine the percent of macrophages to eat FITC-IgG beads. The result indicated that FN significantly increased the phagocytosis to IgG beads of wild-type macrophages (vehicle vs. FN: 5.4 ± 1.40% vs. 7.6 ± 1.39%) (Figure 3(b) and Suppl. Figure 1(b)). Rac1 and Cdc42 have been determined to regulate macrophages phagocytosis of IgG-coated particles and *S. typhimurium* [25,26]. To determine whether these GTPases participate in the phagocytosis of *E. coli* and IgG beads, we assayed Rac1/2, and Cdc42 expression in LPS or PGN stimulated macrophages with or without FN. FN significantly increased Rac1 and Cdc42 in stimulated macrophages (Figure 3(c-e)). Those data determined that FN significantly enhance phagocytosis of macrophages to microbes.

### Integrin β1^-/-^ macrophages have significantly decreased innate immune responses to LPS and PGN *in vitro*

Integrin α5β1 binds to fibronectin’s RGD-containing domain to activate cell function [27]. If FN regulates macrophages through integrin β1 signal pathway, integrin β1^-/-^ macrophages will have inadequate innate responses of macrophages, as well as FN will lose an increased innate response of integrin β1^-/-^ macrophages. To examine this hypothesis, the function of integrin β1 in macrophage phagocytosis and phagosome maturation was examined with integrin β1^-/-^ mice. CD29 (integrin β) f/f mice were bred with LysM-Cre transgenic mice to generate integrin β1^-/-^ macrophages. Integrin β1 deletion in peritoneal macrophages from CD29f/f/LysM-Cre mice were confirmed by flow cytometry (data not shown). To ensure the full deletion of integrin β1 macrophages used in our experiments, integrin β1^-/-^ peritoneal macrophages were sorted by FACS.

**Figure 4.**
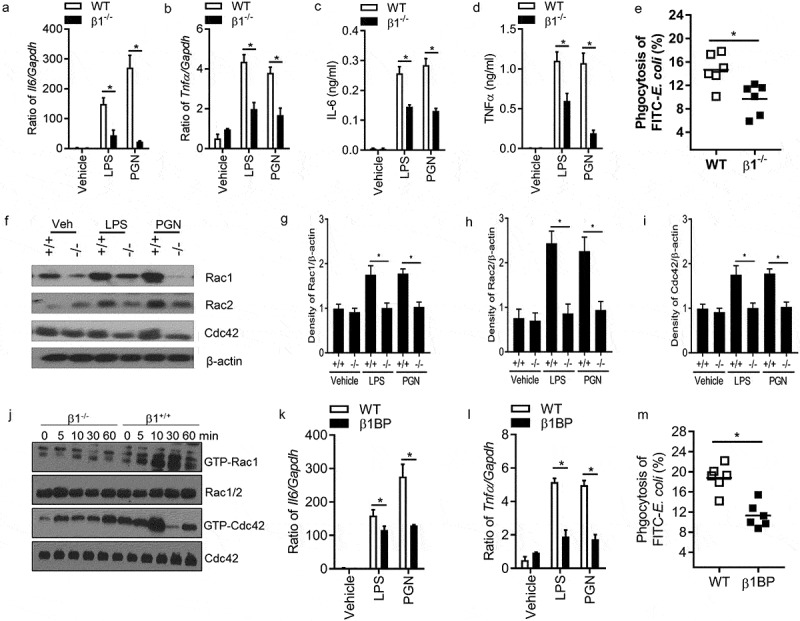
Ablation of Integrin β1 has defective responses to LPS and PGN in macrophages. *Il6* (a) and *Tnfα* (b) mRNA expression were quantified by qPCR after LPS and PGN stimulation in WT and integrin β1^-/-^ macrophages (n = 5, *p < 0.05). IL-6 (c) and TNFα (d) protein level were detected in the supernatant of WT and integrin β1^-/-^ macrophages after LPS and PGN stimulation by ELISA (n = 5, *p < 0.05). Phagocytosis of integrin β1^+/+^ and integrin β1^-/-^ macrophages to FITC conjugated *E. coli* was examined by FACS (e). Peritoneal macrophages from integrin β1-deficient and WT mice and FITC-labeled bacteria opsonized with normal mouse serum were mixed in suspension for 30 min at 37°C at a ratio of 1:100 (cell/bacteria). Phagocytosis was assessed by detecting the MFI of the cells after quenching by FACS. (n = 6, *p < 0.05). Total Rac1, Rac2 and Cdc42 protein in integrin β1^-/-^ and integrin β1^+/+^ peritoneal macrophages were analyzed by western blot after LPS and PGN stimulation (f), and Rac1 (g), Rac2 (h) and Cdc42 (i) densities were quantified by comparing with β-actin with ImageJ software. (n = 3, *p < 0.05). The activity of Rac1, Rac2 and Cdc42 protein in integrin β1^-/-^ and integrin β1^+/+^ peritoneal macrophages were also analyzed by western blot after LPS stimulation (j). *Il6* (k) and *Tnfα* (l) *mRNA* expression of macrophages were quantified by qPCR with and without integrin β1 blocking peptide after LPS and PGN stimulation (n = 5, *p < 0.05). Phagocytosis of macrophages to FITC conjugated E. coli was examined with/without integrin β1 blocking peptide by FACS (m). (n = 5, *P < 0.05). Statistical comparisons among treatment groups were performed by randomized-design two-way ANOVA and followed by unpaired Student’s t-test for two groups using Prism software (Graph Pad Inc., La Jolla, CA). Statistical significance was defined as a P value of less than 0.05.

Integrin β1 is known phagocytic receptors that promote innate responses of macrophages [17]. Other integrins including integrins αMβ2 (CD11b/CD18) and αxβ2 (CD11c/CD18) are also determined to induce complement-mediated phagocytosis, the pro-inflammatory cytokines (IL-6 and TNF-α) production of integrin β1^-/-^ macrophages were at first examined after LPS and PGN stimulation. Integrin β1^-/-^ macrophages have significantly defective IL-6 and TNF-α production (Figure 4(a-d)) after LPS and PGN stimulation. We compared the phagocytosis of *E. coli* by integrin β1^-/-^ and control peritoneal macrophages. Integrin β1^-/-^ macrophages phagocytosed significantly less *E. coli* (∼20% reduction) (Figure 4(e)). These results indicate that pro-inflammatory cytokine production and efficient phagocytosis of *E. coli* critically require integrin β1 signal, and suggest that FN enhances macrophage phagocytosis through integrin β1.

Rho GTPase including Rac1, Rac2, and Cdc42 regulates phagocytosis and the maturation of phagosomes of macrophage to damaged cells [28]. Reduced levels of Rac1, Rac2, and Cdc42 also are a critical mechanism that integrin β1^-/-^ dendritic cells (DCs) fail to efficiently prime T lymphocytes [16]. These results suggested that integrin β1 regulates macrophage phagocytosis through enhancing the expression of Rac1, Rac2, and Cdc42 in macrophages. To test whether FN promotes phagocytosis of macrophages through binding integrin β1 and regulating the expression of Rho GTPase, we investigated Rac1, Rac2, and Cdc42 expression in integrin β1^-/-^peritoneal macrophages and integrin β1^+/+^ macrophages with or without LPS and PGN by western blot. The results showed that integrin β1^+/+^ macrophages expressed sufficient levels of Rac1, Rac2, and Cdc42 before and after stimulation with LPS and PGN, however, there is significantly decreased expression of total Rac1, Rac2, and Cdc42 proteins in integrin β1^-/-^ macrophages stimulated with or without LPS and PGN (Figure 4(f-i)). Integrin β1^-/-^ macrophages also have significantly decreased activities of Rac1 and Cdc42 proteins compared with WT macrophages after LPS stimulation (Figure 4(j)). Our results suggest that there was defective phagocytosis in integrin β1^-/-^ macrophages, which of mechanism may be through deletion of integrin β1 reducing expression of Rac1, Rac2, and Cdc42. More importantly, administration of integrin β1 blocking peptide also has significantly defective *Il6, Tnfa* expression and phagocytosis of macrophages after LPS and PGN stimulation (Figure 4(k-m)), which suggest that integrin β1 may have important clinical significance.

### FN-integrin β1 signaling significantly promotes phagocytosis and phagosome maturation macrophages through co-operating with TLR2/4 receptors in macrophages

**Figure 5.**
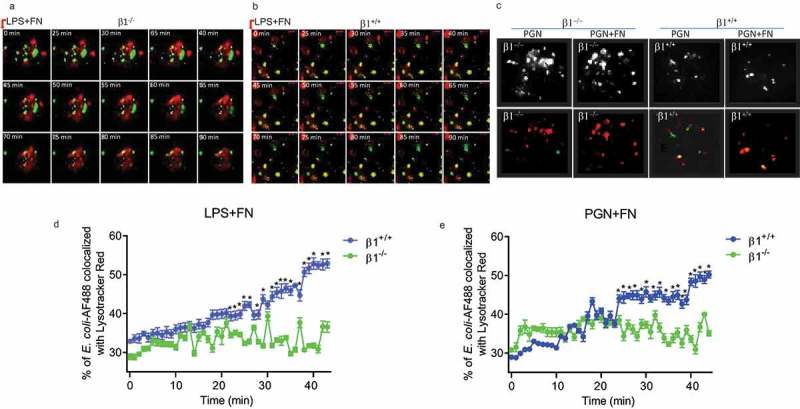
FN has an impaired phagosome/lysosome fusion of macrophages in integrin β1^-/-^ macrophages. Confocal images showing the colocalization of AF488-labeled *E. coli* with Lysotracker Red in integrin β1^-/-^ macrophages (β1^-/-^) and integrin β1^+/+^ macrophages(β1^+/+^). To observe the effect of FN plus LPS in regulating phagosome/lysosome fusion of integrin β1^+/+^ (a) and integrin β1^-/-^ (b) macrophages, Integrin β1^+/+^ and integrin β1^-/-^ peritoneal macrophages pre-adhered to coverslips were preloaded with Lysotracker Red and incubated with opsonized AF488-*E. coli* at a ratio of 1:50 (cell/bacteria), and taken the images at 0, 25, 30, 35, 40, 45, 50, 55, 60, 65,70, 75, 80, 85 and 90 min at 37°C with LPS (1ug/ml) plus FN (1 ug/ml) by laser confocal microscopy. Since macrophage phagosomes are often formed at about 30 min after macrophage phagocytosis to bacteria, for the observation of FN plus PGN (c), the colocalization images of phagosome with lysosome were taken only at 30 min with PGN (1ug/ml) alone and PGN(1ug/ml) plus FN(1ug/ml) in integrin β1^-/-^ macrophages and integrin β1^+/+^ macrophages (lower panel in C), and the colocalization white field images of phagosome with lysosome were also taken in the same time (upper panel in C). In addition, to determine the kinetics of phagosome maturation, the co-localization of AF488-labeled *E. coli* with Lysotracker Red was examined by laser confocal microscopy (LSM 710, Carl Zeiss) at every minute interval from starting the macrophages of integrin β1^-/-^ or integrin β1^+/+^ mice mixed with FITC-conjugated *E. coli* and adding FN plus LPS (d) or FN plus PGN (e) up to 50 min. The percentage of *E. coli* -containing phagosomes that co-localized with Lysotracker (fluorescence overlay) was quantitatively analyzed using the co-localization dialogue of Metamorph software version 7.0 (Universal Imaging, a subsidiary of Molecular Devices) by randomly scanning > 10 cells in each test group in 3 independent experiments (d and e). (n = 3, *p < 0.05). Statistical comparisons among treatment groups were performed by randomized-design two-way ANOVA and followed by unpaired Student’s t-test for two groups using Prism software (Graph Pad Inc., La Jolla, CA). Statistical significance was defined as a P value of less than 0.05.

Macrophages phagocytosis of bacteria form phagosomes and further mature into phagolysosomes, which is the fusion process of a phagosome with a lysosome during phagocytosis. Therefore, we use co-localizing phagosomes, and lysosomal markers identify phagosome maturation. LysoTracker Red may selectively conjugate late endosomes and lysosomes, and co-localizes with lysosome-associated membrane proteins (LAMP) [29,30]. Macrophage phagosomes are formed at about 30 min after macrophage phagocytosis to bacteria. Therefore, we used AF488-labeled *E. coli* in matured phagosome to co-localize with Lysotracker Red over time from 25 to 90 minutes for FN plus LPS in integrin β1^+/+^ and integrin β<^-/-^ macrophages under confocal fluorescence microscopy (Figure 5(a,b)). We also compared the macrophage phagosome formation between integrin β1^+/+^ and integrin β1^-/-^ macrophages in adding FN plus PGN at 30 min after mixing bacteria and macrophages under confocal fluorescence microscopy (Figure 5(c)). The confocal microscopic images showed that the co-localization between this AF-488-*E. coli* and Lysotracker Red two-colored markers were significantly increased in integrin β1^+/+^ macrophages compared with integrin β1^-/-^ macrophages when treated with FN plus LPS or FN plus PGN (Figure 5(a-c)). Further, the co-localization between this AF-488-*E. coli* and Lysotracker Red two-colored markers were quantitated by Metamorph software [31,32]. Whereas the co-localization AF-488-*E. coli* with Lysotracker red obtained 42% or 47% in integrin β1^+/+^ macrophages at 30 min treated with LPS or PGN plus FN, but co-localization was only around 30% treated LPS or PGN in integrin β1^-/-^ macrophages (Figure 5(d,e)). There is significantly decreased overlay of AF488-conjugated *E. coli* with Lysotracker Red in peritoneal macrophages observed at the different time point as shown in integrin β1^-/-^ macrophages treated with LPS or PGN plus FN (Figure 5(d,e)). These results suggest that FN promotes phagosome maturation through integrin β1 signaling.

**Figure 6.**
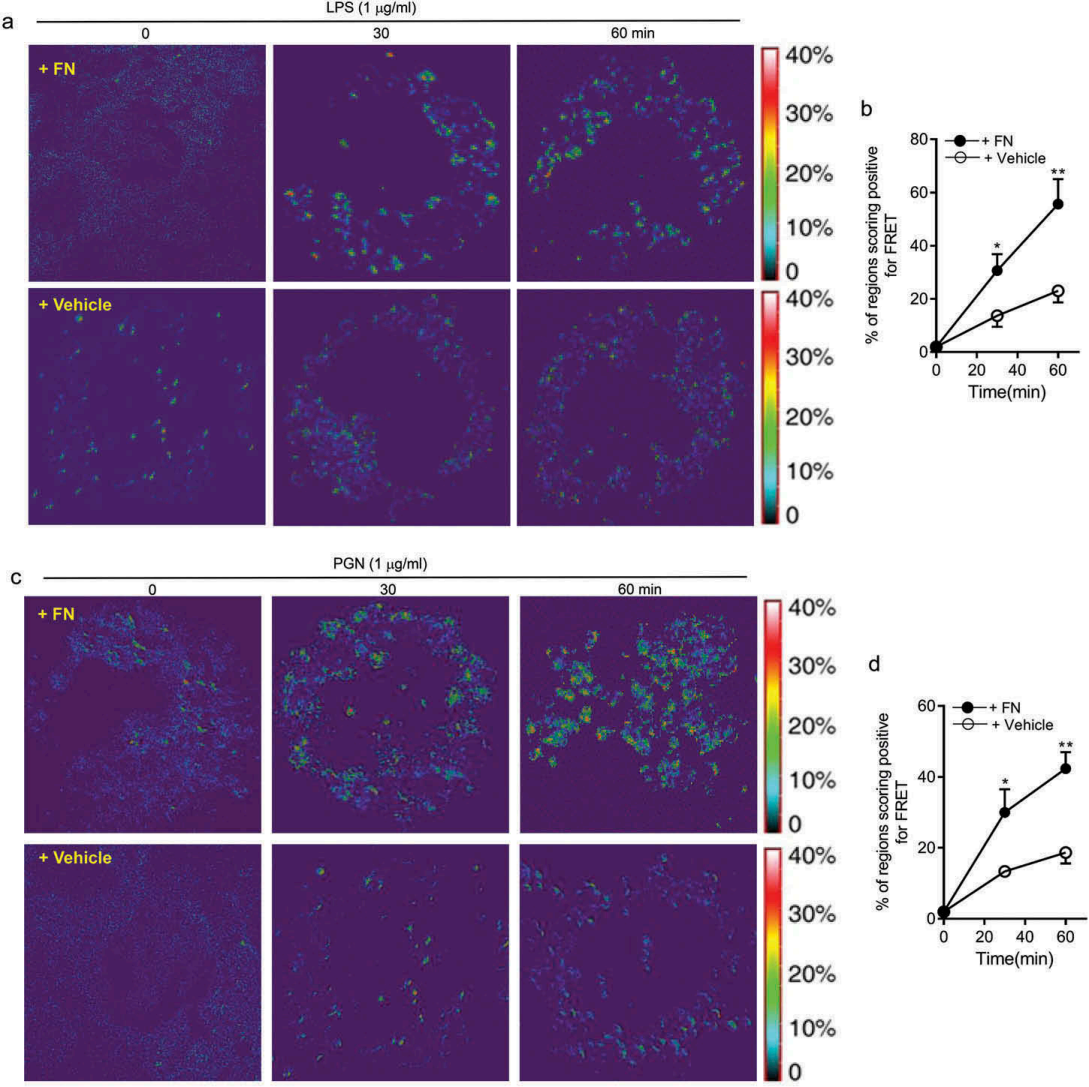
FN significantly promotes innate immune responses of macrophages through interaction of FN-integrin β1 signal and TLR2/TLR4 signal. FRET assay measuring the access of an integrin β1-associated donor fluor AF488 or FITC-TLR4 or TLR2-localized with fluor AF594 or PE-integrin β1 was used to quantify the association of TLR4 or TLR2 and integrin β1 following LPS (a and b) or PGN (c and d) with or without FN. After stimulation of LPS or PGN for 0 min and a chase of 30, 60 min at 37°C, FRET was measured by laser confocal microscopy using the FRET Acceptor Bleaching program, which verifies the donor molecule’s loss of intensity when in close proximity to an acceptor molecule, before and after selective bleaching of the acceptor. Representative images from FRET assay showing the association of AF488-labeled TLR4 Ab or TLR2Ab with AF594-labeled integrin β1 Ab in peritoneal macrophages (a and c). Comparison of the percentage of FRET-positive regions and FRET efficiency between integrin β1 and TLR4 or TLR2 with fibronectin or without in peritoneal macrophages (b and d). The energy transfer efficiency was quantified, as described in Materials and Methods. The percentages of FRET-positive regions were quantified by randomly counting over 10–15 areas within one cell and counting > 10 cells in each tested group in three or more independent experiments. Data are mean ± SE from triplicate samples and are representative of three independent experiments. ∗ p < 0.01; ∗∗, p < 0.05. Statistical comparisons among treatment groups were performed by randomized-design two-way ANOVA and followed by unpaired Student’s t-test for two groups using Prism software (Graph Pad Inc., La Jolla, CA). Statistical significance was defined as a * P value of less than 0.05 and . as a ** P value of less than 0.01.

LPS and PGN activated macrophages through their surface receptors TLR2/4 to regulate innate responses including phagocytosis maturation and pro-inflammatory cytokine production of peritoneal macrophages. To determine the mechanistic role of FN in phagocytosis, phagosome maturation and pro-inflammatory cytokine production of peritoneal macrophages, we applied FRET assays under confocal microscopy to quantitated integrin β1 signal and TLR4 or TLR2 interaction. FRET assay measuring the access of an integrin β1-associated donor fluor AF488 or FITC-TLR4 or TLR2-localized with fluor AF594 or PE-integrin β1 was used to quantify the association of TLR4 or TLR2 and integrin β1 following LPS or PGN with or without FN. The FRET, the energy transfer efficiency, represents the increased percentage of donor intensity after acceptor photobleaching. Our results indicated a rapid increase in FRET signal in a time-dependent manner in peritoneal macrophages with FN stimulation, but there is a dramatically reduced FRET signal in macrophages following LPS and PGN treatment without FN (Figure 6(a-d)). These data demonstrate that FN promotes phagocytosis, phagosome maturation and pro-inflammatory cytokine responses in peritoneal macrophages to LPS and PGN via a TLR2/4 and integrin β1 signal interaction.

## Discussion

Direct or indirect pulmonary injury triggers an acute inflammatory response in lung characterized by increased expression and deposition of ECM components such as collagen and FN [33,34]. Our data also showed that lung macrophages in lung tissues have significantly increased FN expression after LPS treatment compared with PBS treatment stained with anti-fibronectin Ab by confocal microscopy (Figure 1(a)). Those results suggested that FN was involved the regulation of inflammation and innate immunity. Although the function of FN deposited after lung injury is not clear, the effects of FN in regulating activation, migration, proliferation, and differentiation of the cells suggests the potential role in the initiation and maintenance of the inflammatory response *in vivo*. By binding to integrin family receptors on the cell surface, fibronectin transduced the interaction signals between immune cells and non-immune cells into the cell and promotes inflammation by facilitating neutrophils recruitment and cytokine release. Our results also reveal a role of FN in innate immune responses of macrophages through cooperating with TLR2 or TLR4 signaling, which supports the initial hypothesis of FN’s function in the innate immune system.

When macrophages meet with microbial pathogens, the surfaces receptor (such as TLRs) of macrophages was initial by PAMPs of microbial pathogens and Damage-associated molecular patterns (DAMPs) of injured tissues and activate the innate immune response to release various type of proinflammatory cytokines, such as IL-1β, IL-6, TNFα and etc, to recruit neutrophils and further activate adaptive immune system. FN not only provide a structural framework for tissues as scaffolding but also regulates the behavior of the cells contact with it. Interactions between FN and cells affect the activation, migration, adhesion, proliferation, and differentiation of the cells [35]. FN may provide a binding site for the colonization of microorganisms in the host [36,37]. The enhanced activation of LPS-TLR4 and PGN-TLR2 signaling by FN suggests that FN might participate in the initial immune responses against pathogens as an integral part of PRRs [38]. Additionally, our results indicate that (1) FN enhanced the peritoneal macrophages responses to PAMPs and increased the pro-inflammatory cytokines, such as TNF-α and IL-6 released; (2) FN promotes phagocytosis of *E. coli*, and FN enhances FcR-mediated phagocytosis of IgG beads; (3) FN also helps phagosome maturation of macrophages induced by LPS and PGN signaling. Those further demonstrate that FN is essential in the innate immune response.

Another critical function of macrophages is phagocytosis to microbial pathogens in innate immune response. It has been reported that extracellular matrix molecule, such as Mindin, may serve as an opsonin to enhance phagocytosis to bacteria and virus and promote inflammatory cell recruitment. Therefore, as an extracellular protein FN may also strengthen phagocytosis of macrophages to microbes. FN may have multiple functions in innate immune response to microbial infection. Our results also determined that FN promote a phagosome/lysosome fusion of macrophages (Figure 5) or phagosome maturation through regulating GTPase small protein expression (Figure 4), which of regulation can be directly or indirectly. Phagosome maturation requires drastic remodeling of the phagosome membrane and contents. Phagosome maturation of macrophages depends on both microtubule and the actin cytoskeleton [39,40]. It was reported that mature phagosomes were often surrounded by an actin-rich cytoplasm [41]. It has been demonstrated that endosomes and lysosomes are propelled by the formation of actin comet tails [42]. Furthermore, β-actin was detectable as a component of fully formed phagosomes [43], and numerous β-actin-binding proteins, including annexins, α-actinin, and coronin, associate with phagosomes [44]. FN can bind integrin β. In this study, we show that FN could not promote pro-inflammatory cytokines and phagocytosis in integrin β1^-/-^ macrophages (Figure 4). The reduction of Rac1/2 in integrin β1^-/-^ macrophages may impair the process of phagosome/lysosome fusion through a decreased phagosome-associated actin polymerization. These results suggest that FN-integrin β1-mediated signaling plays an important role in modulating the release pro-inflammatory cytokines, phagocytosis, and phagosome maturation through increasing total Rac1/2 and Cdc42 protein expression and activities in macrophages.

It has been reported that integrin α5β1 binds to the RGD-containing domain of FN to activate cell function [27]. Integrin β1 is a known phagocytic receptor that promotes innate responses of macrophages [17]. Although FN can bind to other integrins including αMβ2 (CD11b/CD18) and αxβ2 (CD11c/CD18), which also induce complement-mediated phagocytosis, integrin β1 is highly expressed in macrophages and DCs [45]. Integrin β1 could share several alpha chains to form functional integrins including α1, α2, α3, α4, etc. on the cell surface. However, it was reported that integrin α5β1 mainly expresses in macrophages, and FN also strongly binds to integrin α5β1 [46]. Therefore, in this study, we used integrin β1 deficient mice to investigate the function of FN-integrin β1 signal in regulating the pro-inflammatory cytokine production and phagocytosis of macrophages.

Our results determined that FN-integrin β1 signaling cooperate with TLR2/TLR4 receptors to promote innate immune responses of macrophages. Although it has been reported that FN may directly interact with TLR4 to activate macrophages, our FRET assay results indicate that FN enhances the activation of macrophages by PAMPs through integrin β1 signaling and TLR2/4 signaling cooperation. Additionally, FN could not promote phagocytosis, pro-inflammatory cytokines release and phagosome maturation of macrophages in integrin β1-deficient macrophages, which strongly support that FN enhances pro-inflammatory cytokines, phagocytosis and phagosome maturation of macrophages through integrin β1 signaling. FN significantly increased the expression of Rac1 and Cdc42 of macrophages activated with LPS and PGN, as well as, the expression of Rac1/2 and Cdc42 in LPS and PGN-stimulated integrin β1^-/-^ macrophages were impaired. These results indicate that FN induced signaling is as important as the TLRs signaling pathways which are well-defined. Based on these results, we were the first to demonstrate that FN-mediated integrin β1 recognition of PAMPs represents a secondary stimulation that is essential for the activation of macrophages. Further studies that elucidate FN-mediated signaling pathways of macrophage activation and differentiation will reveal the role and mechanisms of macrophages and FN in the initiated and resolved of the inflammation.

## Abbreviations

ECM: Extracellular matrix
FN: Fibronectin
TLR: Toll-like receptor
LPS: Lipopolysaccharide
LTA: Lipoteichoic acid
PGN: Peptidoglycan
IL-6: Interleukin 6
TNF-α: Tumor necrosis factor-α
Rac1/2: Ras-related C3 botulinum toxin substrate 1/2
Cdc42: Cell division control protein 42 homolog
ARDS: Acute Respiratory Distress Syndrome
ICU: Intensive Care Unit
IL-1β: Interleukin 1β
IL-8: Interleukin 8
PMN: Polymorphonuclear neutrophils
PAMP: Pathogen-associated molecular pattern
PRRs: Pattern-recognition receptors
MBL: Mannose-binding lectin
P38 MAP kinase: P38 mitogen-activated protein kinases
NF-κB: Nuclear factor kappa-light-chain-enhancer of activated B cells
DAMP: Damage-associated molecular pattern
NLRP3: NLR family pyrin domain containing 3
GTPase: Guanosine triphosphate hydrolase
CD29: Cluster of differentiation 29
PE: Phycoerythrin
FITC: Fluorescein isothiocyanate
MAN: Mannan
ZYM: Zymosan
IP: Intraperitoneal injection
IHC: Immunohistochemistry
BMMϕ: Bone marrow-derived macrophages
FACS: Flow cytometry
FcγR: Fc gamma receptor
FITC: Fluorescein isothiocyanate
qPCR: Real-time quantitative RT-PCR
ELISA: Enzyme-linked immunosorbent assay
PBS: Phosphate-buffered saline
FRET: Fluorescence resonance energy transfer
ECL: Enhanced chemiluminescence
SRBC: Sheep red blood cell
LAMP: Lysosomal-Associated Membrane Protein
DCs: Dendritic cells
