# Supplementary material for: Fibronectin (FN) cooperated with TLR2/TLR4 receptor to promote innate immune responses of macrophages via binding to integrin β1
Source: Virulence. 2018 Oct 13;9(1):1588–600. doi: 10.1080/21505594.2018.1528841 (PMC7000207; doi:10.1080/21505594.2018.1528841)
Supplement: Supplemental Material [file kvir-09-01-1528841-g0001.pptx]

## Slide 1
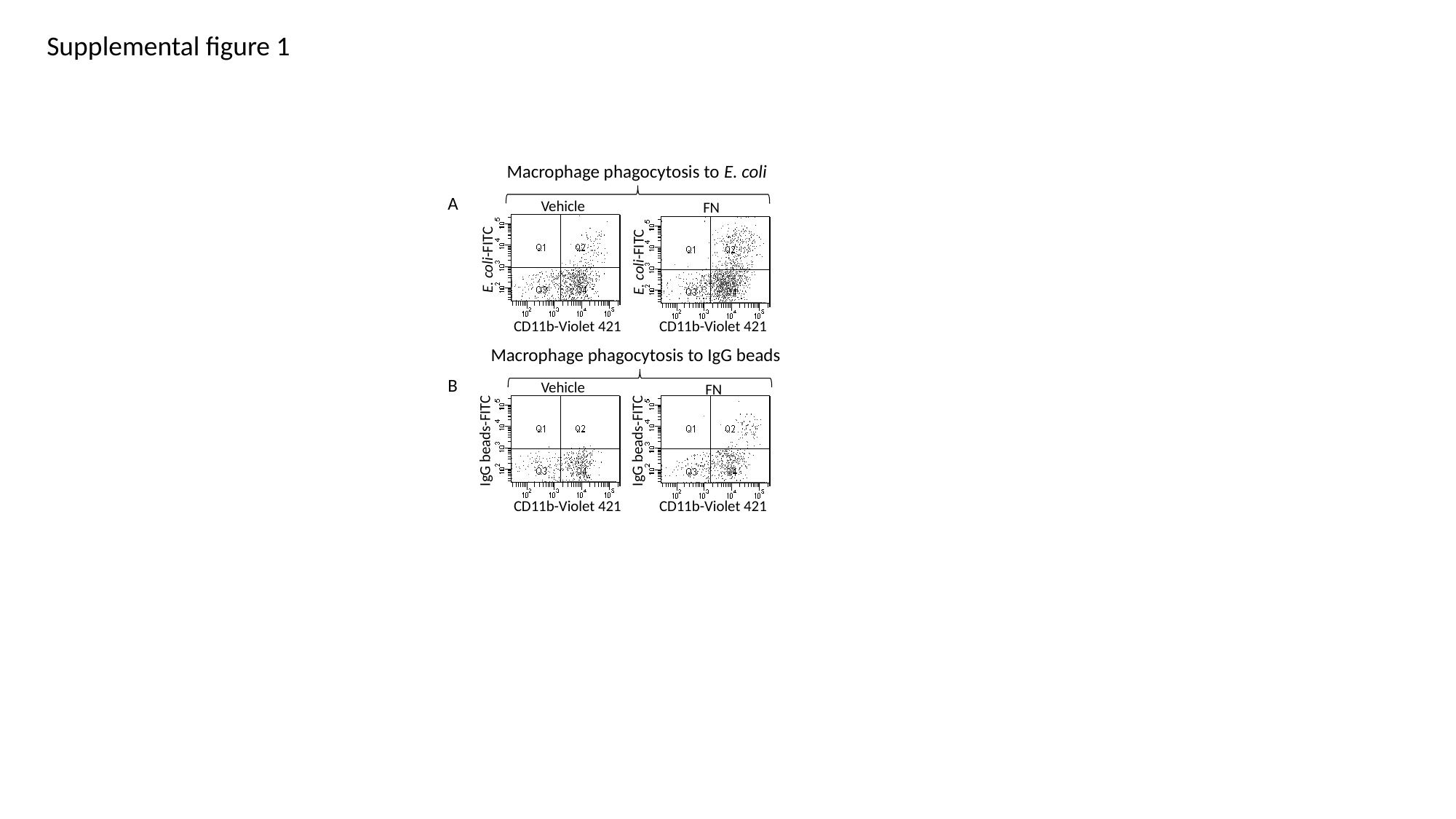

Supplemental figure 1
Macrophage phagocytosis to E. coli
A
Vehicle
FN
E. coli-FITC
E. coli-FITC
CD11b-Violet 421
CD11b-Violet 421
Macrophage phagocytosis to IgG beads
B
Vehicle
FN
IgG beads-FITC
IgG beads-FITC
CD11b-Violet 421
CD11b-Violet 421
